# Lower trust in artificial intelligence than physicians coexists with high willingness to engage in artificial intelligence‐assisted prostate cancer management among active surveillance patients

**DOI:** 10.1002/bco2.70262

**Published:** 2026-09-22

**Authors:** Eric S. Chai, Kaitlynn Abraham, Riley Hanes, Adam Williams, Howard Wolinsky, Oleksandr N. Kryvenko, Radka Stoyanova, Frank J. Penedo, Archan Khandekar, Bruno Nahar, Mark L. Gonzalgo, Dipen J. Parekh, Sanoj Punnen

**Affiliations:** ^1^ School of Medicine University of Missouri‐Kansas City Kansas City Missouri USA; ^2^ Sylvester Comprehensive Cancer Center University of Miami Miller School of Medicine Miami Florida USA; ^3^ Dr. Kiran C. Patel College of Osteopathic Medicine Nova Southeastern University Davie Florida USA; ^4^ Brady Urology Institute Johns Hopkins School of Medicine Baltimore Maryland USA; ^5^ Miller School of Medicine University of Miami Miami Florida USA; ^6^ Freelance Medical Journalist at “The Active Surveillor” USA; ^7^ Department of Pathology and Laboratory Medicine University of Miami Miller School of Medicine Miami Florida USA; ^8^ Department of Radiation Oncology University of Miami Miller School of Medicine Miami Florida USA

**Keywords:** active surveillance, artificial intelligence, prostate cancer, treatment, trust

## Abstract

**Objectives:**

This study aimed to examine the sentiment for artificial intelligence (AI) among prostate cancer (PCa) patients on active surveillance (AS) and to evaluate how that sentiment relates to patient willingness to engage with AI.

**Materials and Methods:**

We conducted a 23‐question online survey (November–December 2025) distributed through an AS platform. Analyses were restricted to 110 respondents with PCa on AS. Outcomes included trust ratings (10‐point Likert scale) for physicians versus AI, responses to hypothetical AI management recommendations and perceived concerns and facilitators of trust. Statistical analyses included the Wilcoxon signed‐rank tests, the McNemar tests and Fisher's exact tests.

**Results:**

Median trust in urologists (8 [IQR 7–9]) exceeded that for AI (6 [IQR 5–7]), with smaller differences for radiologists and pathologists compared with diagnostic AI (all *p* ≤ 0.001). Despite lower trust in AI, most respondents anticipated improved PCa outcomes with AI integration. High willingness to consider AI recommendations was also observed. Only 4.6% (*n* = 5) of respondents outright rejected AI recommendation for treatment and only 3.7% (*n* = 4) rejected AI recommendation for AS, with all others willing to consider AI recommendations. No respondents fully accepted AI recommendation for treatment without additional verification, whereas 2.8% (*n* = 3) fully accepted AI recommendations for continued surveillance. Primary concerns included AI errors (57.3%) and lack of human oversight (48.2%).

**Conclusions:**

Among AS patients, lower trust in AI compared to physicians coexists with a high optimism for AI and willingness to incorporate AI recommendations into decision‐making. This discordance suggests that behavioural adoption of AI‐assisted care may precede full patient confidence.

## 1 INTRODUCTION

Artificial intelligence (AI) has been heralded as a game changer for medicine, destined to “disrupt every part of health and health care delivery”.^1^ Alongside these expectations, the number of studies examining the implementation of AI in healthcare has exponentially increased in recent years.^2^ However, many of these studies focus on pathology‐specific AI efficacy. Understanding of how patient perspectives relate to willingness to engage with AI remains poor and research on specific patient populations' perspectives regarding AI has also been limited. Ethical considerations raise an important question of how comfortable patients are with AI and how that may influence participation in the projected physician‐patient‐AI care of the future.^1^, ^3^, ^4^

Across cancer studies, AI has demonstrated a strong ability to aid in reading imaging, interpreting biopsies, developing new drugs and assisting operatively.^5^ Notably, in 2021, Paige Prostate became the first FDA‐authorized AI software designed to detect prostate cancer (PCa). Since then, AI has proven particularly useful in synthesizing imaging, pathology, and clinical data into predictive models for prognosis and risk stratification.^6^, ^7^, ^8^, ^9^, ^10^, ^11^, ^12^

For both biopsy and MRI analysis in PCa, AI has appeared to be at least as good as, if not superior, to human pathologists and radiologists in comparative studies, particularly with regard to consistency and speed.^6^, ^7^, ^8^, ^9^, ^13^ AI‐based predictive models for PCa metastasis, recurrence and prognosis as well as a model for PCa stage progression in active surveillance (AS) have been trialled.^10^, ^11^ AI predicted treatment response and prognosis have the potential to better guide future treatment decisions.^9^ There have already been promising findings with machine learning recommendations for brachytherapy, and efforts to develop models for personalized PCa treatment recommendations beyond radiation have been fruitful. ^6^, ^12^, ^14^

Patient autonomy and shared decision‐making encourage patient awareness and acceptance of the methods used to direct their care.^3^, ^4^ The efficacy of AI in healthcare may remain constrained without meaningful patient engagement. It is thus important to understand not only patient perspectives regarding AI but also how they respond to the incorporation of AI in patient‐facing care.^15^

There has been limited study on how patient perceptions of AI relate to willingness to engage with AI. The viewpoints of AS PCa patients in particular also remain poorly understood. Given the advancements in AI for PCa diagnostics and treatment planning, it is important to assess patient response to avoid inappropriate AI implementation. AS patients are unique for their longitudinal relationship with physicians and continued assessment of progression resulting in recurrent decision points. The repeated diagnostic testing in AS, which continuously informs decisions to continue surveillance or pursue treatment, increases patients' exposure to AI‐driven processes or recommendations over time. This study aimed to evaluate perceptions towards AI and openness to engage with AI recommendations among men with PCa undergoing AS.

## 2 METHODS

### 2.1 Study design

From November to December of 2025, our team distributed a 23‐question online REDCap survey. Distribution was through “The Active Surveillor”, a Substack newsletter, run by medical journalist Howard Wolinsky, who has been on AS for more than 15 years. The site serves as a community for AS patients with PCa and also attracts an audience of their family members and those who have undergone treatment. The newsletter has an approximate subscriber base of 2700 individuals, and we sent out a recruitment post every 2 weeks for 2 months. Additionally, one‐time promotions of the survey were posted through the AnCan and Active Surveillance Patients International (ASPI) platforms. All survey responses were voluntary, and 149 responses were collected by the end of the 2‐month period (estimated 5.5% response rate). No demographic or other identifying data was collected, and Institutional Review Board approval from the University of Miami was granted in October of 2025.

Our first survey question distinguished the respondents' self‐reported association with PCa. For the present study, we examined only the 110 respondents with PCa who reported being on AS.

### 2.2 Survey instrument

Our survey evaluated several domains. Respondents were first asked about their AI knowledge and usage patterns to establish a baseline of technological familiarity. Next, trust in human doctors versus AI was assessed using Likert 10‐point scales. Urologists were rated against AI in healthcare, radiologists against trained AI in MRI analysis and pathologists against trained AI in prostate biopsy analysis. A 0 corresponded to “Do not trust at all”, and a 10 corresponded to “Completely trust”. Internal consistency of AI‐related trust items was high (Cronbach's *α* = 0.92), which supports the Likert scales' reliability as a composite measure of trust. Differences in trust rating were calculated by subtracting AI trust from physician trust. To better gauge how AI trust would project into patient decision‐making, scenarios were given in which AI recommended treatment or AS after a doctor had confirmed eligibility for both. Individuals could choose from several responses ranging from complete rejection to careful consideration to full acceptance of the AI's recommendation. We also included categorical questions inquiring about patient concerns regarding AI and factors that would increase their trust in AI. We included options commonly raised in AI discourse, and respondents were encouraged to select as many as they felt applied to them. Lastly, we had respondents indicate what they believed the effect of AI use would be on PCa outcomes, with options ranging from significantly worse to significantly better.

### 2.3 Statistical analysis

**FIGURE 1:**
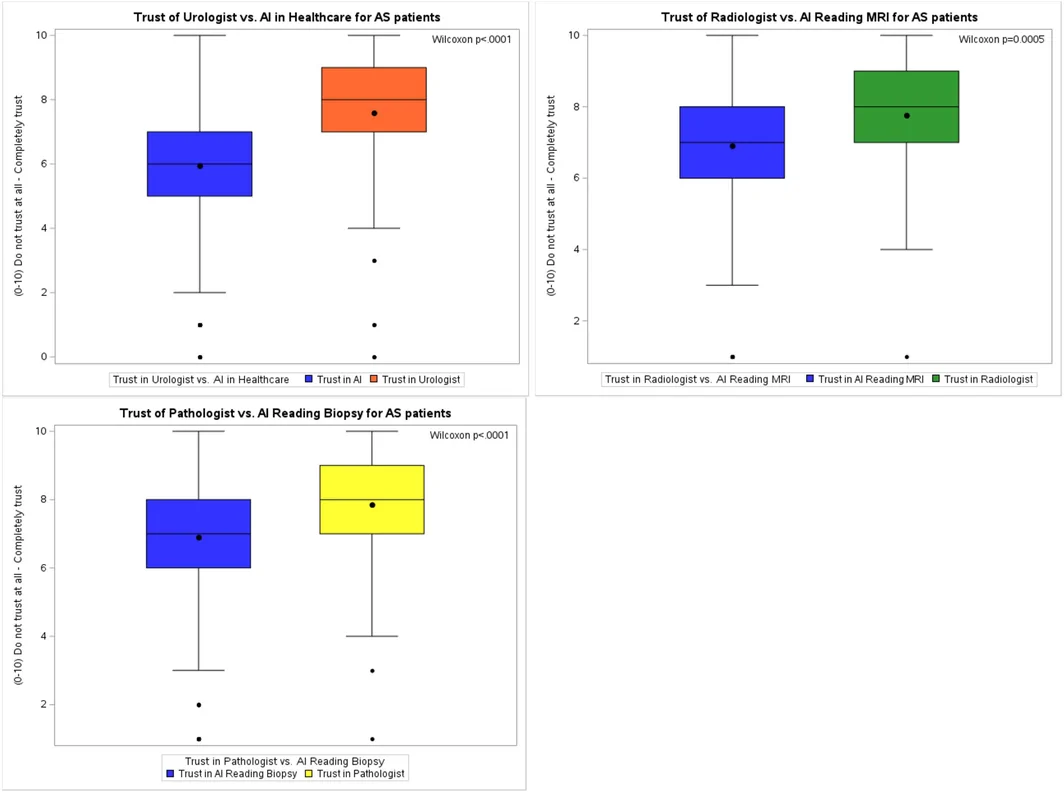
Patient trust in providers versus artificial intelligence in prostate cancer management. Boxplots demonstrating trust ratings on a 10‐point Likert scale for urologists versus AI in healthcare, radiologists versus trained AI in prostate MRI interpretation and pathologists versus trained AI in prostate biopsy interpretation among patients undergoing active surveillance for prostate cancer. Median trust ratings for physicians exceeded those for AI across all comparisons. Differences were assessed using Wilcoxon signed‐rank testing.

**FIGURE 2:**
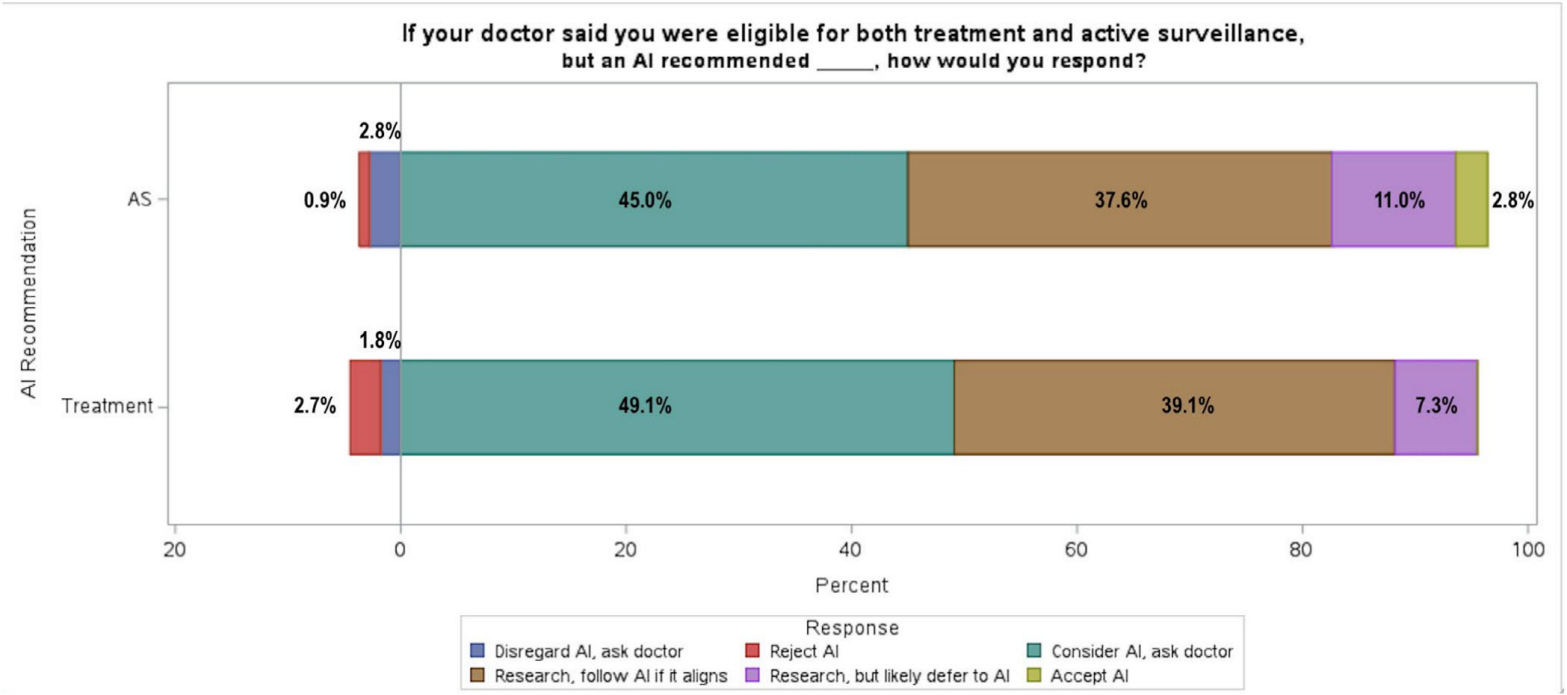
Patient responses to artificial intelligence prostate cancer management recommendations. Distribution of responses to hypothetical AI recommendations for either continued active surveillance or definitive treatment among patients eligible for both management strategies. Most respondents reported willingness to consider AI recommendations with either additional physician consultation or independent verification. Differences in response distributions were assessed using exact McNemar testing.

**FIGURE 3:**
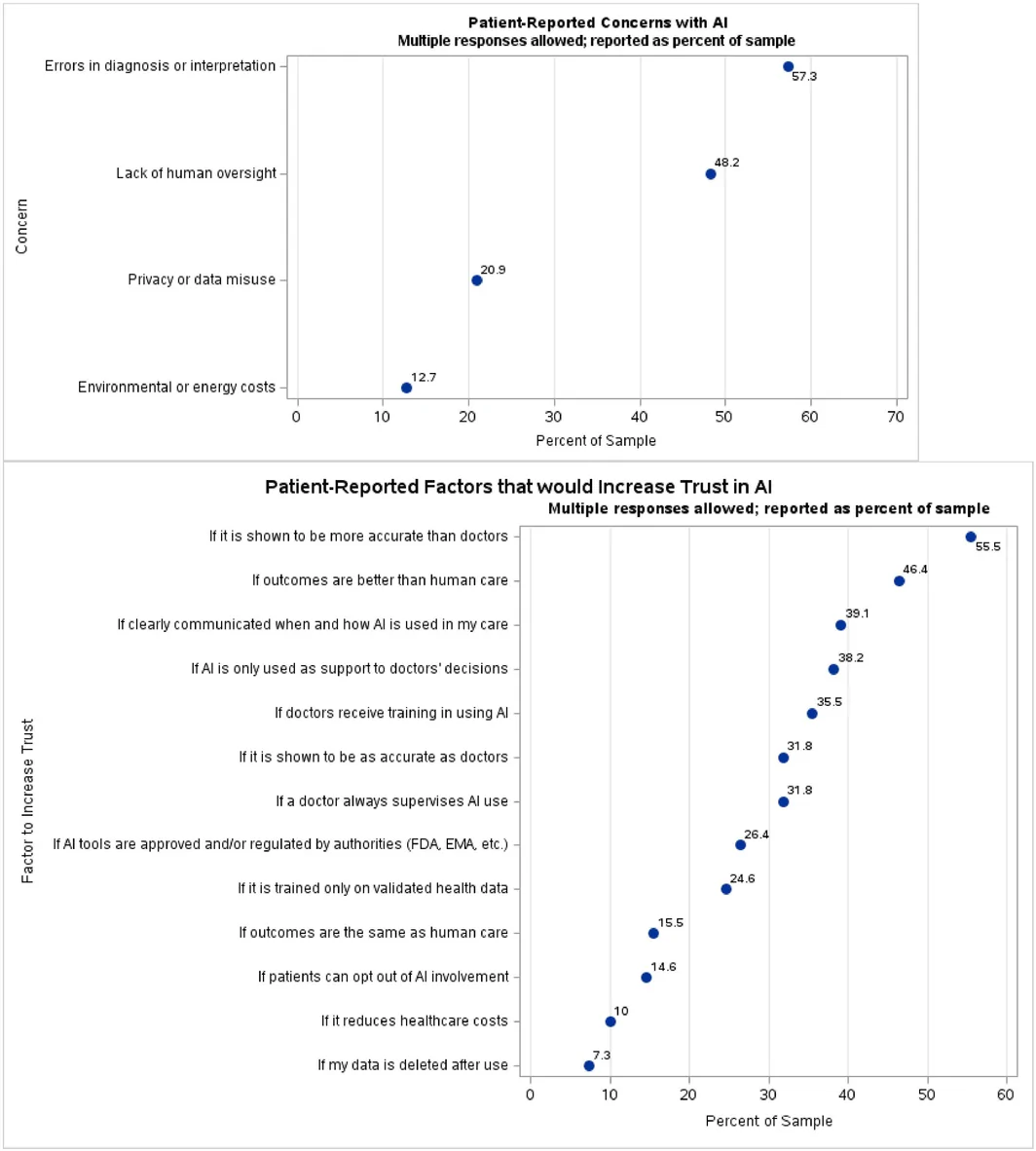
Patient concerns regarding artificial intelligence and factors associated with increased trust in artificial intelligence. Top panel demonstrates the proportion of respondents selecting specific concerns regarding AI use in prostate cancer management. Bottom panel demonstrates factors respondents reported would increase their trust in AI‐assisted medical care. Respondents were permitted to select multiple response options.

**FIGURE 4:**
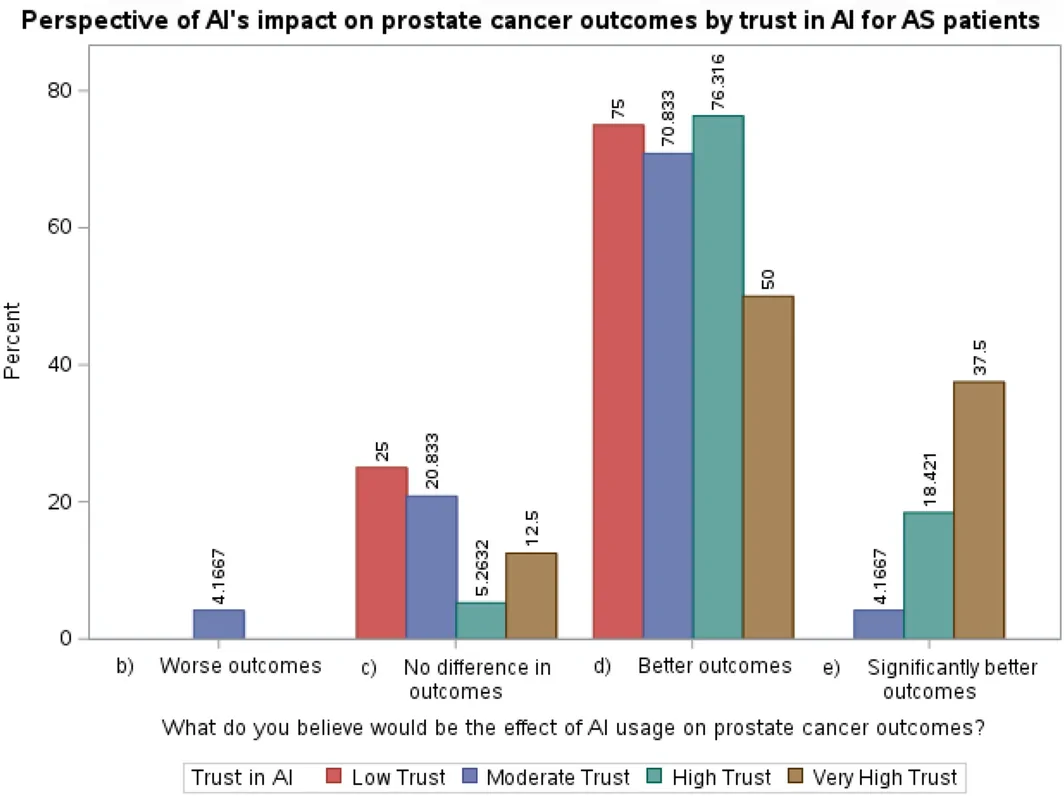
Patient perspectives regarding AI impact on prostate cancer outcomes stratified by AI trust level. Distribution of respondent perceptions regarding the expected impact of AI integration on prostate cancer outcomes according to baseline trust in AI for healthcare. Higher AI trust was associated with greater optimism regarding AI‐assisted prostate cancer care (Fisher's exact test *p* = 0.0257).

Many of our observations used descriptive statistics examining counts and percentages to characterize our cohort. Medians and interquartile range (IQR) were used to describe the trust ratings for providers and AI. Differences in trust between providers and AI were assessed using the Wilcoxon signed‐rank test for paired ordinal data (Figure 1). For Figure 2, exact McNemar tests compared differences in response to AI for a recommendation of AS versus treatment. Figure 3 was purely descriptive of selection rate within the overall cohort. Fisher's exact tests were performed for categorical comparisons, including trust stratification subgroup analysis (Figure 4 and Table S1). All statistical tests were two‐sided, and a *p*‐value <0.05 was considered statistically significant.

## 3 RESULTS

### 3.1 Cohort characteristics

Most respondents had moderate familiarity with AI. 82.8% (*n* = 91) classified themselves as fairly or a little knowledgeable about AI (Table 1). Consistent with this familiarity, most reported usage of AI: 43.1% (*n* = 47) reported occasional use and 39.5% (*n* = 43) reported greater‐than‐occasional use. However, use of AI specifically for health or medical purposes was lower; 25.5% (*n* = 28) reported occasional use with 19.1% (*n* = 21) and 32.7% (*n* = 36) reporting minimal or no use, respectively. ChatGPT (*n* = 55, 50%) and Google Gemini (*n* = 35, 31.8%) were the most commonly used AI programs within our sample.

**TABLE 1:** Artificial intelligence knowledge and usage patterns among patients undergoing active surveillance for prostate cancer.

	Active surveillance
	(*n* = 110)
How knowledgeable are you about Artificial Intelligence (AI)?	
I don't know what it is	0
I've heard of it but don't know much	8 (7.3%)
I know a little about it	49 (44.6%)
I'm fairly knowledgeable	42 (38.2%)
I'm very knowledgeable	9 (8.2%)
I'm an expert (professional capacity)	2 (1.8%)
How often do you use AI in daily life (e.g., Siri, ChatGPT, Google Assistant)?	
I've never used AI	8 (7.3%)
I've tried it once or twice	11 (10.1%)
I use it occasionally	47 (43.1%)
I use it often	15 (13.8%)
I use it regularly	11 (10.1%)
It's part of my daily routine	17 (15.6%)
Have you ever used AI for medical or health‐related purposes?	
No, never	36 (32.7%)
Yes, once or twice	21 (19.1%)
Occasionally	28 (25.5%)
Often	10 (9.1%)
Regularly	15 (13.6%)
Which AI programs do you choose to use? (Select all that apply)	
None	18 (16.4%)
ChatGPT	55 (50%)
Google Gemini	35 (31.8%)
Claude	7 (6.4%)
Microsoft Copilot	21 (19.1%)
Grok	13 (11.8%)
Perplexity	12 (10%)

*Note*: Self‐reported familiarity with AI, frequency of AI usage in daily life and healthcare contexts and commonly used AI platforms among survey respondents undergoing active surveillance for prostate cancer.

### 3.2 Trust in physicians versus AI

Trust ratings of providers and AI were generally moderate to high among respondents (Figure 1). The 10‐point scale defined 0 as *do not trust at all* and 10 as *completely trust* in the context of prostate cancer management. The median trust rating of urologists was an 8 (IQR 7–9) compared with 6 (IQR 5–7) for AI in healthcare. For prostate cancer MRI and biopsy analysis, radiologists and pathologists were both given a median rating of 8 (IQR 7–9) compared with 7 (IQR 6–8) for trained AI in each diagnostic modality.

Median trust difference of 2 [IQR 0–3] on a 10‐point scale (*p* < 0.001) indicated a meaningful preference for urologists over AI. Median rating differences for radiologists (0 [IQR 0–2]; *p* = 0.0005) and pathologists (0 [IQR 0–3]; *p* < 0.001) against AI were present but minimal.

Stratification of our cohort by trust for AI in healthcare revealed 12 (11%) reported low trust (0–3 rating), 51 (46.8%) reported moderate trust (4–6 rating), 38 (34.9%) reported high trust (7–8 rating) and 8 (7.3%) reported very high trust (9–10 rating).

### 3.3 Willingness to accept AI recommendations

When presented with a hypothetical scenario in which respondents are eligible for both treatment or AS, no statistically significant difference in response was observed when AI recommended one over the other (Figure 2). However, most respondents were open to considering AI's recommendation. Only 3.7% (*n* = 4) of individuals would not consider AI's recommendation for AS, and 4.5% (*n* = 5) outright rejected AI's recommendation for treatment. Furthermore, when stratified by trust, 8.3% of those with low trust in AI, compared with 2% with moderate and 2.6% with high trust in AI, would not consider AI's recommendation for AS. When AI recommended treatment, 25% (*n* = 3) of those with low AI trust would not consider it, and 8.3% (*n* = 1) would completely reject it (Figure S1).

Notably, no respondents were willing to fully accept AI's treatment recommendation, whereas 25% of those with very high AI trust (and 2.8% of the total cohort) were willing to do so for an AS recommendation. Furthermore, considering AI's recommendation but subsequently consulting a provider or performing additional personal research were the most common responses.

### 3.4 Patient concerns regarding AI and factors that may increase trust in AI

Respondents were given a short list of possible concerns regarding AI use in PCa management. A greater proportion of those with higher AI trust reported no concerns (*p* = 0.0043) (Table S1). However, the most commonly selected concerns were errors in diagnosis/interpretation (57.3% of the cohort) and lack of human oversight (48.2%) (Figure 3). Concerns regarding privacy/data misuse (20.9%) and environmental or energy costs (12.7%) were less common.

In line with these concerns, 55.5% of respondents said that they would have greater trust in AI if it was shown to be more accurate than doctors. About 46.4% said demonstrating better outcomes with AI would increase their trust. However, this dropped to 31.8% for demonstrating equal accuracy and 15.5% for achieving equal outcomes to human doctors.

A large proportion of respondents also reported implementing transparency around AI usage (39.1%), training doctors on using AI (35.5%) and limiting AI to supportive (38.2%) or constantly supervised (31.8%) roles as factors that would improve trust. Reduced healthcare costs (10%) and data deletion (7.3%) were the least‐selected factors for improving AI trust in medical care among our respondents.

For those with AI trust ranging from low to high, selection rates for concerns and factors that may increase trust appeared fairly consistent (Table S1). Small sample sizes for the low and very high trust categories limit interpretability, but no major differences in distribution were observed in our sample.

### 3.5 Optimism for AI

Despite concerns regarding AI, the vast majority of respondents reported optimism for PCa outcomes with AI implementation (Figure 4). Those with higher trust in AI were more likely to report greater optimism regarding the impact of AI (Fisher's exact *p* = 0.0257). However, even for those with low trust in AI, 75% (*n* = 8) predict better outcomes from AI's impact.

## 4 DISCUSSION

Our cohort of AS patients had a mostly moderate self‐reported level of AI familiarity with low usage of AI in health‐related contexts. Trust ratings for both AI and physicians centred around the upper moderate to high range of trust; however, urologists received the highest trust ratings relative to AI. Differences in trust between radiologists/pathologists and trained AI in diagnostics appeared present but clinically insignificant.

Despite differences in trust for AI, willingness to consider AI management recommendations, whether that be AS or treatment, was high. Few respondents disregarded AI recommendations, even for those with low AI trust. Most took AI recommendations into consideration with further independent or physician‐assisted confirmation. No respondents fully accepted an AI recommendation for treatment without further verification unlike the several that fully accepted an AS recommendation. The risk asymmetry between initiating treatment and continuing AS may contribute to more careful deliberation on recommendations for treatment. The high levels of consideration for both AI recommendations indicate openness to including AI as part of overall management decision‐making.

This openness to engaging with AI recommendations even for those with low AI trust can be explained by the specific factors contributing to lower trust in AI. The top AI concern among respondents was accuracy and efficacy. Another substantial concern was lack of oversight with over a third of respondents being more willing to trust AI if it were used purely as a support tool supervised by physicians. In our hypothetical scenarios, most respondents considered AI recommendations with additional independent or physician‐assisted verification. Collaboration between humans and AI in shared decision‐making likely mitigates concerns regarding accuracy or rogue autonomy that would otherwise cause apprehension in accepting AI integration. Thus, trust in AI may lag behind openness to adoption of AI‐assisted care. As AI operates in a collaborative role, data regarding efficacy may be gathered and patient exposure to AI systems may increase, advancing patient trust.

Another explanation for differences in trust is the highly valued interpersonal relationship between providers and PCa patients.^16^ Urologists' direct interaction and longitudinal relationships with patients likely contribute to higher trust relative to radiologists, pathologists and AI. Diagnostic evaluation by an unseen AI versus an unseen physician may be harder to distinguish relative to patient‐facing roles. A 2023 Western European study on MRI analysis of PCa found substantially greater trust in radiologists than in AI compared to our findings.^17^ The discrepancy may be due to shifts in perspective as AI pervasiveness has increased over time and differences in digital literacy or cultural between cohorts. In the hypothetical scenarios, the most common response was to consider AI's recommendation and then consult their physician regarding it. This highlights the remaining importance of the physician–patient relationship particularly in the early stages of AI integration where trust in AI does not align with engagement with AI. Over a third of respondents reported physician training in AI use would increase their willingness to trust AI. During early adoption, patients may strongly rely on physicians to clarify how they should use and interpret AI recommendations.

Given the reliance patients may have on physicians during early stages of AI adoption, open dialogue regarding how AI fits in patient care is important. Although there is currently no clear consensus regarding whether providers' use of AI tools must be disclosed to patients, respondents noted clear communication regarding AI use in their care as the top factor that would increase their trust in AI after factors relating to accuracy/efficacy.^18^ Transparency regarding early AI integration in medical care can serve as exposure that may promote patient comfort with AI in future expanded roles.

Unlike prior studies on patient perceptions of AI, relatively few of our respondents found data or reduced costs to be factors that would influence their opinion on AI.^15^ Less concern regarding data privacy may reflect differences in understanding and tolerance regarding data privacy in recent times. As AI tools and discourse about online data have become more mainstream, reduced data privacy has widely become recognized as inherent to the current digital age.^19^ Additionally, unlike the general patients surveyed by Moy et al., PCa patients are more likely to be insured privately and live in higher socioeconomic areas.^20^, ^21^ PCa patients may therefore put lower emphasis on financial considerations.

Despite lower trust in AI compared to physicians, most respondents believe AI integration will have positive effects on PCa outcomes. A significantly lower number of respondents selected they would be more willing to trust AI if it has equivalent accuracy or outcomes to humans compared to superior accuracy or outcomes. Comparable efficacy to humans appears to be a baseline expectation such that only superior efficacy warrants recalibration of perceptions. With these high expectations for AI, it appears that patients are receptive to exploring AI‐assisted approaches even with underlying apprehensions regarding trustworthiness.

## 5 IMPLICATIONS

AS of PCa represents a high frequency diagnostic and decision environment that is uniquely sensitive to AI‐assisted care. The vast majority of surveyed AS patients, regardless of their current trust in AI, have great optimism for the impact of AI on PCa care.

Despite lower trust in AI than physicians, AS patients remained highly willing to incorporate AI recommendations into decision‐making. The lower trust in AI appears secondary to concerns regarding physician oversight and validated accuracy. Utilizing AI as an assistive device in decision‐making with human collaboration may bridge the trust deficit that may otherwise have induced hesitation in engaging with fully autonomous AI. Our findings suggest that behavioural adoption of AI in clinical care may precede full patient confidence.

## 6 LIMITATIONS

Our survey was distributed through an online site and newsletter focused on AS in PCa. Each survey question and response was completely voluntary; however, most respondents completed all questions. Our cohort likely represents a digitally engaged, health‐literate subset of AS patients, which may lead to overestimating AI acceptance. These findings may therefore reflect perspectives of early adopters rather than the broader prostate cancer population. However, this population may be particularly relevant to early phases of AI integration. Howard Wolinsky serves as head of the distribution newsletter, cofounder of ASPI/AnCan AS groups and was included as a co‐author in this study, introducing additional selection bias. Demographic information (including age, race and socioeconomic status) was not collected. This limits generalizability, and results should be interpreted as observations of a limited group of AS PCa patients.

For questions regarding AI concerns and factors to increase trust, respondents could select as many of the listed concerns as desired; however, unlisted concerns/factors or further details regarding selected options could not be determined. Additionally, we are unable to determine individual respondents' interpretation of hypothetical scenarios or rating scales, which may differ from one another. The survey instrument was not previously validated and was developed specifically for this study with input from prostate cancer providers and patients, which limits the interpretability of Likert and other responses.

## 7 CONCLUSION

Physicians, particularly those in patient‐facing roles, remain more trusted than AI in PCa care. However, AS patients are highly optimistic for AI integration and demonstrate willingness to incorporate AI recommendations into their decision‐making. Despite concerns regarding errors and a lack of human oversight, openness to AI engagement across all levels of trust suggests patient‐facing integration is feasible before full trust in AI technologies is achieved.

## AUTHOR CONTRIBUTIONS

Conceptualization: Eric S. Chai and Sanoj Punnen. Data curation: Adam Williams. Formal analysis: Adam Williams. Methodology: all authors. Writing—original draft: Eric S. Chai. Writing—review and editing: all authors. Supervision: Sanoj Punnen.

## CONFLICT OF INTEREST STATEMENT

The authors declare no competing interests.

## Supporting information


**Figure S1.** Patient responses to artificial intelligence management recommendations stratified by artificial intelligence trust level. Responses to hypothetical AI recommendations for continued active surveillance (top panel) or definitive treatment (bottom panel) stratified according to respondent baseline trust in AI for healthcare. Most respondents across all trust categories reported willingness to consider AI recommendations, although respondents with lower AI trust were more likely to reject treatment recommendations.

**Table S1.** Concerns regarding artificial intelligence and factors associated with increased trust in artificial intelligence stratified by AI trust level. Distribution of respondent‐selected concerns regarding AI in healthcare and factors reported to increase trust in AI‐assisted care according to baseline trust in AI for healthcare. Respondents were permitted to select multiple response options. P‐values were calculated using Fisher's exact testing.

## Data Availability

The data that support the findings of this study are available on request from the corresponding author. The data are not publicly available due to privacy or ethical restrictions.

## References

[1] Angus DC , Khera R , Lieu T , Liu V , Ahmad FS , Anderson B , et al. AI, health, and health care today and tomorrow: the JAMA summit report on artificial intelligence. Jama. 2025;334(18):1650–1664. 10.1001/jama.2025.18490 41082366

[2] Xie Y , Zhai Y , Lu G . Evolution of artificial intelligence in healthcare: a 30‐year bibliometric study. Front Med. 2025;11:1505692. 10.3389/fmed.2024.1505692 PMC11775008 39882522

[3] Lorenzini G , Arbelaez Ossa L , Shaw DM , Elger BS . Artificial intelligence and the doctor‐patient relationship: expanding the paradigm of shared decision making. Bioethics. 2023;37(5):424–429. 10.1111/bioe.13158 36964989

[4] Cohen IG , Slottje A . Artificial intelligence and the law of informed consent. In: Solaiman B , Cohen IG , editors Research handbook on health, AI and the law Cheltenham (UK): Edward Elgar Publishing Ltd; 2024. 40245217

[5] Najar Najafi N , Hajihassani H , Azimzadeh Irani M . The impact of artificial intelligence on cancer diagnosis and treatment: a review. Cancer Inform. 2025;24:11769351251371273. 10.1177/11769351251371273 PMC12449654 40979169

[6] Arita Y , Roest C , Kwee TC , Paudyal R , Lema‐Dopico A , Fransen S , et al. Advancements in artificial intelligence for prostate cancer: optimizing diagnosis, treatment, and prognostic assessment. Asian J Urol. 2025;12(4):434–444. 10.1016/j.ajur.2024.12.001 PMC12744706 41467190

[7] Pak S , Park SG , Park J , Cho ST , Lee YG , Ahn H . Applications of artificial intelligence in urologic oncology. Investig Clin Urol. 2024;65(3):202–216. 10.4111/icu.20230435 PMC11076794 38714511

[8] Rabaan AA , Bakhrebah MA , AlSaihati H , Alhumaid S , Alsubki RA , Turkistani SA , et al. Artificial intelligence for clinical diagnosis and treatment of prostate cancer. Cancers (Basel). 2022;14(22):5595. 10.3390/cancers14225595 PMC9688370 36428686

[9] Agrawal S , Vagha S . A comprehensive review of artificial intelligence in prostate cancer care: state‐of‐the‐art diagnostic tools and future outlook. Cureus. 2024;16(8):e66225. 10.7759/cureus.66225 PMC11374581 39238711

[10] Nayan M , Salari K , Bozzo A , Ganglberger W , Lu G , Carvalho F , et al. A machine learning approach to predict progression on active surveillance for prostate cancer. Urol Oncol. 2022;40(4):161.e1–161.e7. 10.1016/j.urolonc.2021.08.007 PMC8882704 34465541

[11] Sushentsev N , Rundo L , Abrego L , Li Z , Nazarenko T , Warren AY , et al. Time series radiomics for the prediction of prostate cancer progression in patients on active surveillance. Eur Radiol. 2023;33(6):3792–3800. 10.1007/s00330-023-09438-x PMC10182165 36749370

[12] Naderian S , Soleimanzadeh F , Nikniaz L , Sanaie S , Sadeghi‐Ghyassi F , Samad‐Soltani T . A systematic review of artificial intelligence‐based clinical decision support systems in prostate cancer management. Healthc Technol Lett. 2025;12(1):e70026. 10.1049/htl2.70026 PMC12625777 41262209

[13] Pai RK , Van Booven DJ , Parmar M , Punnen S . A review of current advancements and limitations of artificial intelligence in genitourinary cancers. Am J Clin Exp Urol. 2020;8(5):152–162. PMC7677518 33235893

[14] Tang S , Zhang H , Liang J , Tang S , Li L , Li Y , et al. Prostate cancer treatment recommendation study based on machine learning and SHAP interpreter. Cancer Sci. 2024;115(11):3755–3766. 10.1111/cas.16327 PMC11531952 39223585

[15] Moy S , Irannejad M , Manning SJ , Farahani M , Ahmed Y , Gao E , et al. Patient perspectives on the use of artificial intelligence in health care: a scoping review. J Patient Cent Res Rev. 2024;11(1):51–62. 10.17294/2330-0698.2029 PMC11000703 38596349

[16] Lysø EH , Hesjedal MB , Skolbekken JA , Solbjør M . Men's sociotechnical imaginaries of artificial intelligence for prostate cancer diagnostics: a focus group study. Soc Sci Med. 2024;347:116771. 10.1016/j.socscimed.2024.116771 38537333

[17] Fransen SJ , Kwee TC , Rouw D , Roest C , van Lohuizen QY , Simonis FFJ , et al. Patient perspectives on the use of artificial intelligence in prostate cancer diagnosis on MRI. Eur Radiol. 2025;35(2):769–775. 10.1007/s00330-024-11012-y PMC11782406 39143247

[18] Mello MM , Char D , Xu SH . Ethical obligations to inform patients about use of AI tools. Jama. 2025;334(9):767–770. 10.1001/jama.2025.11417 40690211

[19] Voloch N , Hirschprung RS . Both ends of artificial intelligence impacting privacy: a review of violation and protection. Front Artif Intell. 2026;9:1686454. 10.3389/frai.2026.1686454 PMC12957209 41788612

[20] Grant SR , Walker GV , Guadagnolo BA , Koshy M , Allen PK , Mahmood U . Variation in insurance status by patient demographics and tumor site among nonelderly adult patients with cancer. Cancer. 2015;121(12):2020–2028. 10.1002/cncr.29120 PMC4887084 25917222

[21] Rundle A , Neckerman KM , Sheehan D , Jankowski M , Kryvenko ON , Tang D , et al. A prospective study of socioeconomic status, prostate cancer screening and incidence among men at high risk for prostate cancer. Cancer Cause Control. 2013;24(2):297–303. 10.1007/s10552-012-0108-6 PMC3557724 23224323

