# Supplementary material for: Lower trust in artificial intelligence than physicians coexists with high willingness to engage in artificial intelligence‐assisted prostate cancer management among active surveillance patients
Source: BJUI Compass. 2026 Sep 22;7(9):e70262. doi: 10.1002/bco2.70262 (PMC13595033; doi:10.1002/bco2.70262)
Supplement: Supplementary file 1 [file BCO2-7-e70262-s001.docx]

**Table S1. Concerns regarding artificial intelligence and factors associated with increased trust in artificial intelligence stratified by AI trust level.**
Distribution of respondent-selected concerns regarding AI in healthcare and factors reported to increase trust in AI-assisted care according to baseline trust in AI for healthcare. Respondents were permitted to select multiple response options. P-values were calculated using Fisher’s exact testing.

|  | **Low Trust**  **(n=12)** | **Moderate Trust (n=51)** | **High Trust**  **(n=38)** | **Very High Trust (n=8)** | **p-value** |
| --- | --- | --- | --- | --- | --- |
| **What concerns do you have about AI in healthcare? (Select all that apply)**     No concerns     Privacy or data misuse     Lack of human oversight     Errors in diagnosis or interpretation     Environmental or energy costs | 0  3 (25.0%)  6 (50.0%)  8 (66.7%)    1 (8.3%) | 4 (7.8%)  10 (19.6%)  27 (52.9%)  33 (64.7%)    9 (17.7%) | 8 (21.1%)  8 (21.1%)  17 (44.7%)  20 (52.6%)    4 (10.5%) | 4 (50.0%)  1 (12.5%)  3 (37.5%)  2 (25.0%)    0 | **0.0043**  0.9201  0.7974  0.1493    0.4522 |
| **What would increase your trust in AI for medical care? (Select all that apply)**     Nothing would increase my trust     If it reduces healthcare costs     If it is shown to be as accurate as doctors     If it is shown to be more accurate than doctors     If outcomes are the same as human care     If outcomes are better than human care     If a doctor always supervises AI use     If AI is only used as support to doctors’ decisions     If it is trained only on validated health data     If patients can opt out of AI involvement     If doctors receive training in using AI     If my data is deleted after use     If clearly communicated when and how AI is used in my care     If AI tools are approved and/or regulated by authorities (FDA, EMA, etc.) | 0    0    1 (8.3%)    6 (50.0%)    1 (8.3%)    3 (25.0%)    6 (50.0%)    2 (12.5%)    3 (25.0%)    4 (33.3%)    4 (33.3%)    1 (8.3%)    6 (50.0%)      4 (33.3%) | 1 (1.9%)    7 (13.7%)    18 (35.3%)    29 (56.9%)    9 (17.7%)    22 (43.1%)    15 (29.4%)    17 (33.3%)    9 (17.7%)    6 (11.8%)    16 (31.4%)    6 (11.8%)    18 (35.3%)      11 (21.6%) | 0    4 (10.5%)    15 (39.5%)    22 (57.9%)    6 (15.8%)    22 (57.9%)    11 (30.0%)    19 (50.0%)    12 (31.6%)    6 (15.8%)    16 (42.1%)    0    14 (36.8%)      11 (30.0%) | 1 (12.5%)    0    1 (12.5%)    2 (37.5%)    1 (12.5%)    3 (37.5%)    2 (25.0%)    4 (50.0%)    3 (37.5%)    0    3 (37.5%)    1 (12.5%)    4 (50.0%)      2 (25.0%) | 0.1120    0.3924    0.1268    0.7264    0.8725    0.1954    0.5154    0.1352    0.3870    0.1651    0.7688    0.1863    0.7097      0.7930 |
