# Supplementary material for: Lower trust in artificial intelligence than physicians coexists with high willingness to engage in artificial intelligence‐assisted prostate cancer management among active surveillance patients
Source: BJUI Compass. 2026 Sep 22;7(9):e70262. doi: 10.1002/bco2.70262 (PMC13595033; doi:10.1002/bco2.70262)
Supplement: Supplementary file 2 [file BCO2-7-e70262-s002.docx]

**Figure S1. Patient responses to artificial intelligence management recommendations stratified by artificial intelligence trust level.**
Responses to hypothetical AI recommendations for continued active surveillance (top panel) or definitive treatment (bottom panel) stratified according to respondent baseline trust in AI for healthcare. Most respondents across all trust categories reported willingness to consider AI recommendations, although respondents with lower AI trust were more likely to reject treatment recommendations.
